# Effects of inhaled beclomethasone dipropionate/formoterol fumarate/glycopyrronium on diaphragmatic workload and lung function in uncontrolled asthma: a case report

**DOI:** 10.3389/fmed.2024.1357362

**Published:** 2024-03-05

**Authors:** Antonio Maiorano, Chiara Lupia, Nicola Montenegro, Giuseppe Neri, Andrea Bruni, Eugenio Garofalo, Federico Longhini, Claudia Crimi, Angelantonio Maglio, Alessandro Vatrella, Girolamo Pelaia, Corrado Pelaia

**Affiliations:** ^1^Department of Health Sciences, University “Magna Graecia” of Catanzaro, Catanzaro, Italy; ^2^Department of Medical and Surgical Sciences, University “Magna Graecia” of Catanzaro, Catanzaro, Italy; ^3^Department of Clinical and Experimental Medicine, University of Catania, Catania, Italy; ^4^Department of Medicine, Surgery and Dentistry, University of Salerno, Salerno, Italy

**Keywords:** asthma, triple inhaled therapy, diaphragmatic ultrasound, thickening fraction, ACQ-5

## Abstract

Beclomethasone dipropionate/formoterol fumarate/glycopyrronium (BDP/FF/G) single inhaler extrafine triple therapy is effective for the treatment of uncontrolled asthma. Nevertheless, there is a lack of data about the use of diaphragmatic ultrasonography to monitor adult asthmatics while they are receiving inhaled treatment. We took into consideration a 78-year-old woman complaining of asthma, treated with inhaled corticosteroid/long-acting β_2_-adrenergic agonist (ICS/LABA), characterized by an asthma control questionnaire-5 (ACQ-5) score and a lung function test suggestive of uncontrolled asthma. Moreover, a diaphragmatic ultrasound showed signs of high diaphragm workload. Because of these findings, we proposed to our patient a shift toward triple inhaled therapy with BDP/FF/G, and she underwent a second evaluation after 7 days of treatment. Improvements in the diaphragmatic ultrasound parameters, lung function test, and ACQ-5 score were found. In particular, we detected a reduction of thickening fraction (TF), and a normalization of the other diaphragmatic measures, indicative of a decrease in diaphragmatic workload. To our knowledge, this is the first literature report showing concomitant improvements of both lung function tests and diaphragmatic ultrasonography parameters, observed in an adult patient with uncontrolled asthma after short-term treatment with the single inhaler triple therapy BDP/FF/G.

## Background

Single inhaler extrafine triple therapy with beclomethasone dipropionate/formoterol fumarate/glycopyrronium (BDP/FF/G) is efficacious in the treatment of uncontrolled asthma (1). However, current evidence is quite scarce about the application of ultrasound to follow-up adult asthmatic patients during inhaled therapy. Scioscia et al. evaluated 72 patients complaining of severe uncontrolled asthma using a transthoracic ultrasound (TUS) examination, thus finding thickened and/or irregular pleural lines, associated with either a lack or reduction of the gliding sign (2). Del Colle et al. carried out TUS in three patients using the M-mode pattern, and they did not detect gliding and barcode signs (3). Nevertheless, these observations were based only on TUS, not associated with diaphragmatic ultrasound. Therefore, we herein describe the first reported case of a patient with uncontrolled asthma, treated with the single inhaler extrafine triple therapy BDP/FF/G, who experienced relevant improvements of diaphragmatic function detected by an ultrasound, paralleled by marked amelioration of symptom control and lung function.

## Case presentation

**Figure 1:**
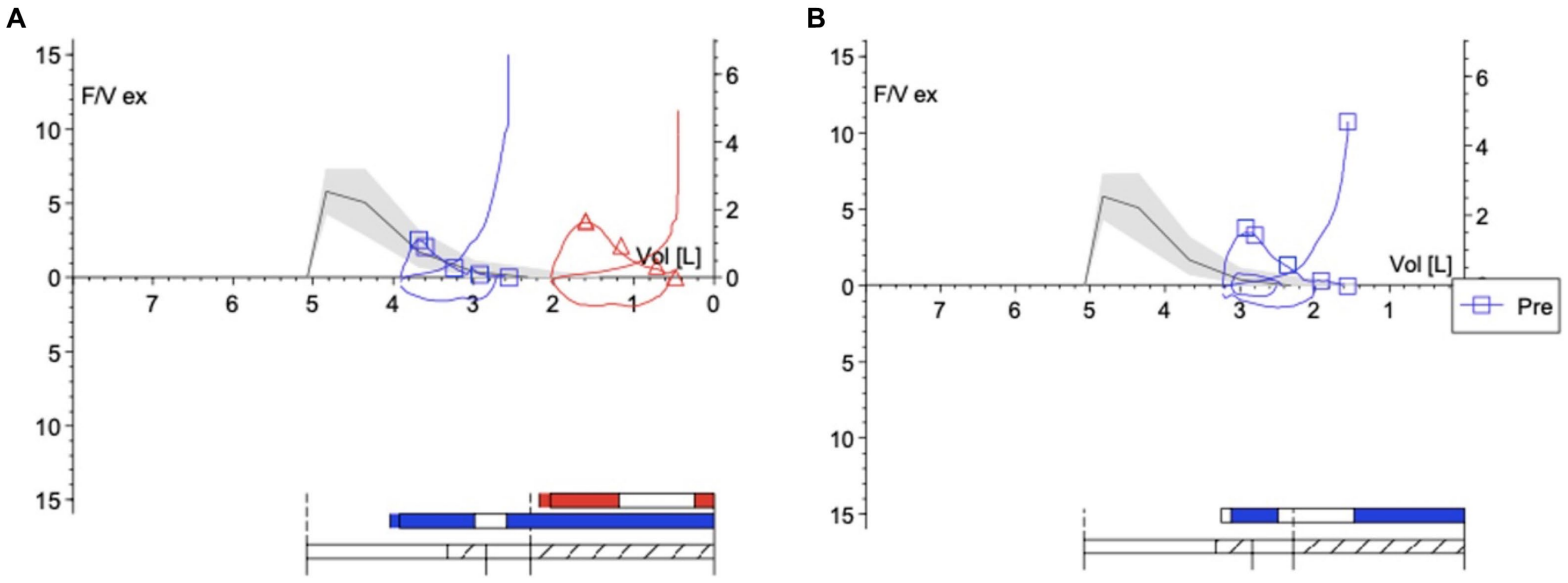
**(A)** Flow–volume curves before BDP/FF/G (blue line for baseline curve and red line for post-bronchodilator test curve), showing the reversibility of airflow limitation. **(B)** Flow–volume curve after BDP/FF/G.

Due to a severe asthma exacerbation, a 78-year-old atopic and never-smoker Caucasian woman was referred to the Pulmonology Unit of “Magna Graecia” University Hospital located in Catanzaro, Italy. The patient was taking high dosages of beclomethasone/formoterol (BDP/FF) inhaled therapy; she correctly performed the therapeutic inhalations and displayed a high degree of adherence to the inhaled treatment. The asthma control questionnaire-5 (ACQ-5) score was 3.8. Vital signs at admission included a body temperature of 36.6°C, arterial blood pressure of 140/90 mmHg, heart rate of 82 beats/min, respiratory rate of 20 breaths/min, and hemoglobin oxygen saturation of 91% in room air. Physiologic chest breathing sounds were diffusely reduced, and relevant wheezing was present. Blood analysis revealed the following values: hemoglobin, 12.3 g/dL; white blood cell count, 10.83 × 10^3^ cells/μL; neutrophils, 57.1% (6,184 cells/μL); eosinophils, 2.8% (303 cells/μL); C-reactive protein level, 17.3 mg/L; erythrocyte sedimentation rate (ESR), 40 mm/h; and total immunoglobulin E (IgE) levels, 852.6 IU/mL. Blood gas analysis at admission (room air/FiO_2_ 21%) yielded the following results: pH, 7.44; PaO_2_, 63 mmHg; PaCO_2_, 35 mmHg; HCO_3_^−^, 24 mmol/L. Echocardiography revealed an estimated pulmonary arterial systolic pressure of 40 mmHg, left ventricular ejection fraction of 61%, reduced right ventricular systolic function, and normal inferior vena cava diameter. Lung function tests performed during ICS/LABA therapy evidenced the following values (Figure 1A): forced expiratory volume in 1 s (FEV_1_), 0.88 L (41% of predicted); forced vital capacity (FVC), 1.34 L (48% of predicted); FEV_1_/FVC ratio, 65.67%; peak expiratory flow (PEF), 2.54 L/s (44% of predicted); residual volume (RV), 2.59 L (113% of predicted); total lung capacity (TLC), 3.93 L (74% of predicted); Motley index (RV/TLC ratio), 65.85%; forced expiratory flow at 75% of FVC (FEF_75_), 2.07 L/s (41% of predicted); forced expiratory flow at 50% of FVC (FEF_50_), 0.66 L/s (39% of predicted); forced expiratory flow at 25% of FVC (FEF_25_), 0.22 L/s (56% of predicted); forced expiratory flow at 25–75% of forced vital capacity (FEF_25–75_), 0.54 L/s (32% of predicted); and total airway resistance (R_tot_), 0.42 kPa x s/L (140% of predicted).

**Figure 2:**
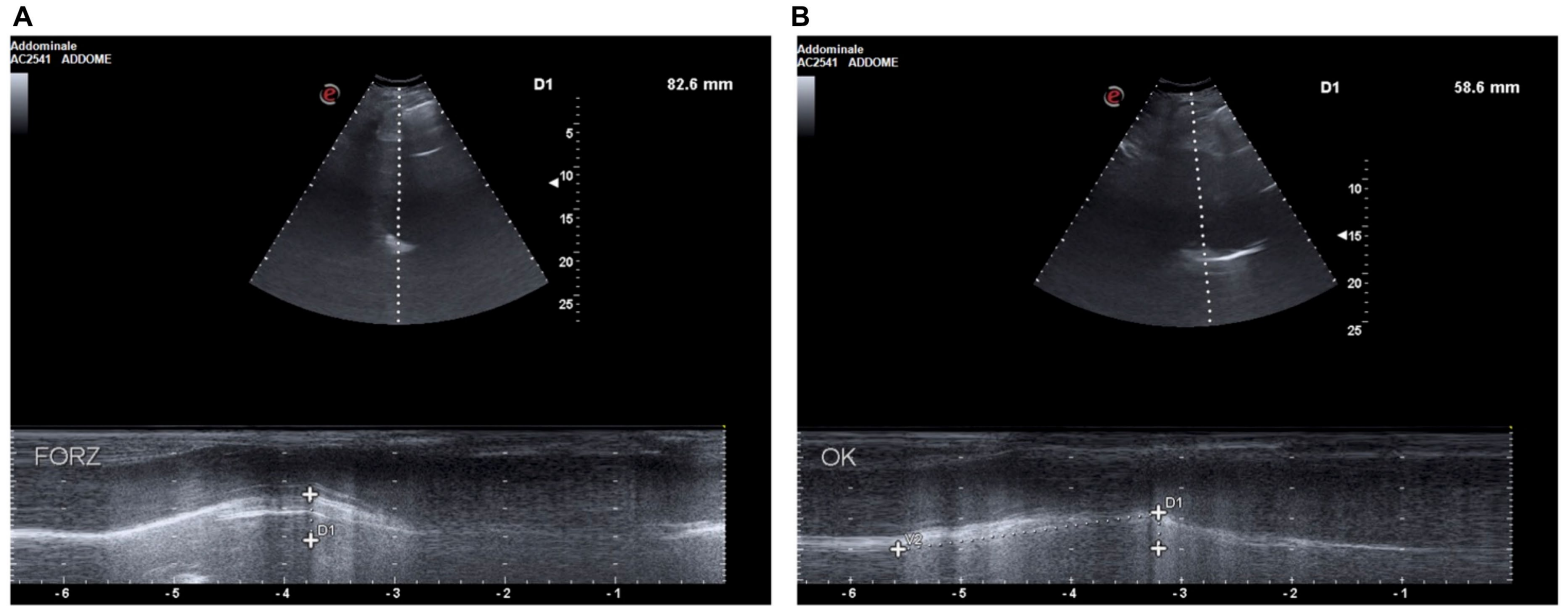
**(A)** D1: deep breathing motion before BDP/FF/G. **(B)** D1: deep breathing motion after BDP/FF/G.

**Figure 3:**
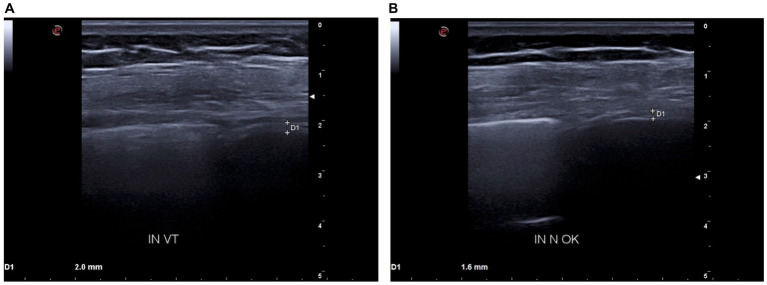
**(A)** D1: tidal volume inspiratory thickness before BDP/FF/G. **(B)** D1: tidal volume inspiratory thickness after BDP/FF/G.

Moreover, baseline diaphragmatic ultrasound evaluation was carried out by a pulmonologist with many years of certified experience. All measurements were made under conditions of spontaneous and resting tidal breathing, during seated and lying down positions, in an abdominal setting, using a 3.5–5-MHz convex ultrasound probe in brightness mode (B-mode) for thickness, and a 7–15-MHz linear probe in B-mode and motion mode (M-mode) for right diaphragmatic shift, respectively (Esaote MyLab XPro30, Esaote S.p.A., Genoa, Italy). The liver was considered as a marker to find an echographic window for the right hemidiaphragm. During the lying down position, the probe was placed between the midclavicular and anterior axillary lines, using the B-mode pattern to select the right hemidiaphragm exploration line. The normal diaphragm contracts and moves to the transducer during inspiration. This is recorded as an upward motion during M-mode, regarded as the diaphragmatic excursion during inspiration, measured on the vertical axis from baseline to the point of maximum height of inspiration. Diaphragmatic excursion was recorded in a frozen image made up of at least three consecutive respiratory cycles, to reduce the measurement error. In the seated position, the diaphragmatic thickness was assessed by B-mode ultrasonography, fixing the linear probe below the phrenicocostal sinus, near the mid-axillary line at the eighth intercostal space, thus identifying the diaphragm as a structure with two parallel echogenic lines, among which there was the hypoechoic diaphragmatic muscle. Its thickness was considered as the distance between the pleural and peritoneal membranes. The measurements were performed at the end of inspiration and expiration during quiet breathing at tidal volume and the end of inspiration after deep breathing. We took into consideration three different breathing cycles. Normal ranges were considered according to those previously described by Boussuges et al. (4, 5) and Zambon et al. (6). We detected the following ultrasound baseline parameters (Figures 2A, 3A): tidal volume excursion, 3.1 cm (normal range: 0.9–2.5 cm); tidal volume inspiratory time, 0.98 s (normal range: 0.5–1.7 s); tidal volume inspiratory velocity, 3.5 cm/s (normal range: 0.7–2.6 cm/s); tidal volume expiratory time, 2.7 s (normal range: 0.4–1.6 s); tidal volume expiratory velocity, 1.2 cm/s (normal range: 0.2–2.8 cm/s); tidal volume duration of motion, 3.8 s (normal range: 1.1–3.4 s); deep breathing excursion, 8.26 mm (normal range: 3.3–7.5 mm); deep breathing inspiratory time, 1.7 s (normal range: 0.4–2.4 s); deep breathing inspiratory velocity, 4.2 cm/s (normal range: 1.5–7.3 cm/s); thickness at end-expiration (FRC), 1.1 mm (normal range: 1.1–2.7 mm); thickness at end-inspiration, 2.0 mm (normal range: 1.3–3.7 mm); tidal volume thickening fraction (TF), 81% (normal range: 30–36%); and thickness at deep breathing, 3.6 mm (normal range: 2.4–5.4 mm).

Because of these findings and clinical worsening, inhaled therapy was changed from BDP/FF to triple fixed-combination BDP/FF/G. After a week of therapy, the ACQ-5 score decreased to 1.2, and follow-up functional lung tests yielded the following results (Figure 1B): FEV_1_, 1.20 L (56% of predicted; +0.32 L/+36.4%); FVC, 1.78 L (63% of predicted; +0.44 L/+32.8%); FEV_1_/FVC ratio, 67.43% (+2.7%); PEF, 3.77 L/s (65% of predicted; +48.4%); RV, 1.48 L (65% of predicted; −1.11 L/−42.9%); TLC, 3.26 L (61% of predicted; −0.67 L/−17.0%); RV/TLC ratio, 45.49 (−30.9%); FEF_75_, 3.30 L/s (65% of predicted; +59.4%); FEF_50_, 1.35 L/s (80% of predicted; +104.5%); FEF_25_, 0.32 L/s (83% of predicted; +45.5%); FEF_75–25_, 0.75 L/s (45% of predicted; +38.9%); and R_tot_, 0.39 kPa x s/L (130% of predicted; −7.1%). Diaphragmatic ultrasound was also performed, thereby evidencing the following measures (Figures 2B, 3B): tidal volume excursion, 1.6 cm (normal range: 0.9–2.5 cm); tidal volume inspiratory time, 1.12 s (normal range: 0.5–1.7 s); tidal volume inspiratory velocity, 1.6 cm/s (normal range: 0.7–2.6 cm/s); tidal volume expiratory time, 1.2 s (normal range 0.4–1.6 s); tidal volume expiratory velocity, 1.6 cm/s (normal range: 0.2–2.8 cm/s); tidal volume duration of motion, 2.3 s (normal range: 1.1–3.4 s); deep breathing excursion, 5.86 mm (normal range: 3.3–7.5 mm); deep breathing inspiratory time, 2.3 s (normal range: 0.4–2.4 s); deep breathing inspiratory velocity, 2.5 cm/s (normal range: 1.5–7.3 cm/s); thickness at end-expiration (FRC), 1.1 mm (normal range: 1.1–2.7 mm); thickness at end-inspiration, 1.6 mm (normal range: 1.3–3.7 mm); tidal volume thickening fraction (TF), 45% (normal range: 30–36%), and thickness at deep breathing, 2.8 mm (normal range: 2.4–5.4 mm). The patient did not experience any adverse effects during treatment with BDP/FF/G.

## Discussion

This case report highlights the improvements in diaphragmatic thickness and motion, detected by ultrasound after 7 days of single inhaler triple therapy with BDP/FF/G (Table 1). Asthma is a chronic obstructive respiratory disease, characterized by bronchial hyperresponsiveness and reversible airflow limitation, caused by airway inflammation and remodeling. In asthma, airway structural changes include subepithelial fibrosis, neo-angiogenesis, smooth muscle thickening, and goblet cell metaplasia/hyperplasia (7). Asthma heterogeneity is expressed by many phenotypes sustained by different endotypes (8). The most common endotypes are included under the umbrella term “type 2 asthma,” referring to either allergic or non-allergic traits, very often associated with airway eosinophilic inflammation (9).

**Table 1:** Diaphragmatic parameters before and after BDP/FF/G treatment.

	Before BDP/FF/G	After BDP/FF/G	Normal values
Excursion (cm)	3.1	1.6	0.9–2.5
Inspiratory velocity (cm/s)	3.5	1.6	0.7–2.6
Expiratory time (s)	2.7	1.2	0.4–1.6
Duration of motion (s)	3.8	2.3	1.1–3.4
Thickening fraction (TF) (%)	81	45	30–36

Over time, small airway inflammation can promote expiratory airflow limitation and dynamic hyperinflation, causing daily exercise intolerance, especially in obese asthmatic patients because of reduced chest wall compliance (10). This finding is well-known in chronic obstructive pulmonary disease (COPD), resulting from expiratory airflow limitation and ventilatory demand, thus contributing to dyspnea and activity impairment (11). These functional limitations can be improved in COPD patients by BDP/FF/G treatment, which decreases air trapping and lung hyperinflation, and also improves lung diffusing capacity (12). Moreover, COPD patients may develop both respiratory and locomotor muscle dysfunctions (13). In hyperinflated patients, the diaphragm works against increased mechanical loads, caused by airflow limitation and geometrical chest changes driven by RV increase (14). Hence, ultrasound may be helpful to evaluate diaphragmatic function in COPD patients, with regard to thickening (15) and motion parameters (16). Moreover, in COPD patients, BDP/FF/G could positively impact the structural changes of the diaphragm (e.g., fiber type transformation, sarcomere injury, and diaphragm atrophy), which contributes to its dysfunction. Thus far, no report has been published about the potential utility of diaphragmatic ultrasound in adult patients with uncontrolled asthma. However, considering the increasing application of ultrasounds in clinical practice, e.g., to evaluate right heart dysfunction (17), presence or absence of pleural effusion (18), COPD abnormalities (19), and liver diseases (20), we decided to study diaphragmatic function using ultrasound in a patient with uncontrolled asthma, before and after therapy with BDP/FF/G. Diaphragmatic ultrasound is not routinely used to follow-up adult patients with severe asthma, before and after inhaled treatments. However, we hypothesized that the occurrence in asthmatic patients of small airway impairment, eventually associated with dynamic hyperinflation (10), could cause diaphragmatic dysfunction (14). The patient evaluated in the present case report was characterized by severe reversible airway obstruction and small airway impairment. This dysfunctional condition was associated with an increased work of the diaphragm, documented by ultrasound and reasonably explained by increased tidal volume motion, expiratory time, motion time, and thickening fraction. The single inhaler double therapy BDP/FF was shifted to single inhaler triple therapy BDP/FF/G, and after a week, the tidal volume TF value was 45%. Other parameters changed and were found to be included within normal ranges. Such data were also reflected by functional respiratory improvements. Therefore, it is reasonable to speculate that the relevant improvement of the diaphragmatic function, which we detected in our patients after BDP/FF/G treatment, was mainly due to a marked decrease in airway resistance, consequently associated with a reduced work of the diaphragm muscle fibers. Furthermore, the patient reported a marked reduction in cough and dyspnea, associated with good control of asthma symptoms, as shown by a decrease in the ACQ-5 score. To the best of our knowledge, this case report represents the first evidence of concomitant improvements involving lung function tests and diaphragmatic ultrasound evaluation, experienced after treatment with single inhaler triple therapy BDP/FF/G by an adult asthmatic patient with uncontrolled asthma.

## Conclusion

This case report highlights for the first time the utility of diaphragmatic ultrasound as a follow-up examination of severe asthmatic patients. In particular, after short-term treatment with the single inhaler triple therapy BDP/FF/G, we detected relevant improvements in functional diaphragmatic performance, paralleled by an important amelioration of lung function. Such a preliminary observation suggests that it could be useful to carry out further studies with the aim of better elucidating the role of diaphragmatic ultrasound evaluation in the management and monitoring of patients with severe uncontrolled asthma. Indeed, although our present considerations are restricted to only one patient, we think that the innovative methodological approach proposed in this case report deserves extensive application in asthmatic patients treated with triple inhaled therapy. The well-known beneficial effect exerted by BDP/FF/G might also depend on the positive role played by this inhaled treatment on the diaphragmatic function of patients with uncontrolled asthma.

## Data availability statement

The raw data supporting the conclusions of this article will be made available by the authors, without undue reservation.

## Ethics statement

The studies involving humans were approved by the Local Ethical Committee of Calabria Region (Catanzaro, Italy; document no. 263 – 23 July 2020). The studies were conducted in accordance with the local legislation and institutional requirements. The participants provided their written informed consent to participate in this study. Written informed consent was obtained from the individual(s) for the publication of any potentially identifiable images or data included in this article.

## Author contributions

AMai: Data curation, Investigation, Methodology, Resources, Visualization, Writing – original draft, Writing – review & editing. CL: Formal analysis, Investigation, Methodology, Visualization, Writing – original draft, Writing – review & editing. NM: Investigation, Methodology, Visualization, Writing – original draft, Writing – review & editing. GN: Supervision, Validation, Visualization, Writing – original draft, Writing – review & editing. AB: Supervision, Validation, Visualization, Writing – original draft, Writing – review & editing. EG: Supervision, Validation, Visualization, Writing – original draft, Writing – review & editing. FL: Supervision, Validation, Visualization, Writing – original draft, Writing – review & editing. CC: Supervision, Validation, Visualization, Writing – original draft, Writing – review & editing. AMag: Supervision, Validation, Visualization, Writing – original draft, Writing – review & editing. AV: Supervision, Validation, Visualization, Writing – original draft, Writing – review & editing. GP: Formal analysis, Investigation, Methodology, Project administration, Supervision, Validation, Visualization, Writing – original draft, Writing – review & editing. CP: Conceptualization, Data curation, Formal analysis, Funding acquisition, Investigation, Methodology, Project administration, Resources, Software, Supervision, Validation, Visualization, Writing – original draft, Writing – review & editing.
